# The mechanism of R2ab-mediated attachment to polystyrene surfaces in *Staphylococcus epidermidis*

**DOI:** 10.1016/j.jbc.2026.113533

**Published:** 2026-09-08

**Authors:** Rahul Yadav, Y. Randika Perera, Radha P. Somarathne, Himanshi Shorie, Soneika Carby, Archana Mishra, Nicholas C. Fitzkee

**Affiliations:** 1 Physical Science Department, University of Arkansas-Fort Smith, Fort Smith, Arkansas, USA; 2 Department of Chemistry, Mississippi State University, Mississippi State, Mississippi, USA

**Keywords:** biofilm, nanoparticles, protein NMR, structure, surface adsorption

## Abstract

The staphylococcal autolysin protein hydrolyzes cell wall peptidoglycans. The R2ab repeats of autolysin guide the catalytic domain by binding to cell wall components and mediate attachment to abiotic surfaces during biofilm formation on polystyrene. Characterizing protein interactions with surfaces is a significant challenge in understanding bacterial attachment. Here, using nuclear magnetic resonance spectroscopy, we determined the solution structure and backbone dynamics of the R2ab domain. We then, by combining nuclear magnetic resonance and calorimetry, identified the interaction mode of R2ab with cell wall components and polystyrene surfaces. The solution-derived R2ab structure reveals interaction surfaces with lipoteichoic acid and peptidoglycan. The binding regions are identified, and docking is used to model their binding to R2ab. R2ab also interacts with polystyrene nanoparticles. Using a novel approach where polystyrene nanoparticles are paramagnetically labeled, we identify the major R2ab-polystyrene interaction interface. R2ab engages polystyrene surfaces *via* a defined C-terminal interface on R2b, as revealed by PRE mapping, isothermal titration calorimetry, and mutational analyses. Polar and charged residues (notably S776 and K779) play key roles in adsorption. These findings support a model in which R2ab acts as a molecular bridge, binding both cell wall components and abiotic surfaces. This highlights its role in early biofilm formation and suggests that disrupting this interaction could reduce *Staphylococcus epidermidis* attachment to abiotic surfaces.

*Staphylococcus epidermidis* is an opportunistic pathogen that predominantly infects immunocompromised individuals, and *S. epidermidis* is a dominant cause of hospital-related infections (1, 2, 3). *S. epidermidis* biofilms present a significant biomedical challenge, and staphylococcal biofilms formed on catheters or implants often result in harder to eradicate chronic infections (4, 5, 6, 7, 8, 9). A high percentage of staphylococcal isolates are resistant to methicillin (10, 11, 12, 13), and biofilm growth has been recognized as a crucial resistance mechanism. Infections involving *S. epidermidis* are very difficult to eradicate, often leading to prolonged antibiotic therapy or surgical removal of the metallic implants and the surrounding infected tissue (14).

Biofilms are multi-cellular bacterial communities anchored to a surface. Bacteria within a biofilm are surrounded by extracellular polymeric substances that reduce bacterial exposure to hostile conditions, including the host immune system (4). The growth of a biofilm on a surface is a multistep process and is regulated by the diverse characteristics of the growth medium, the substratum, and the cell surface. An established biofilm has a defined architecture of microbial cells embedded in extracellular polymeric substances, which provides an optimal environment for exchanging material, including genetic material, between the cells (15). Before this occurs, however, bacteria must attach to and colonize a biotic or abiotic surface. Many proteins are implicated in the attachment of *Staphylococcus* spp. to different surfaces (16, 17), and each protein likely differs in the biophysical properties that mediate surface adhesion. However, a structural model for the initial attachment of a bacterial protein to an abiotic surface has remained elusive.

*S. epidermidis* autolysin (AtlE) is one such protein thought to play a role in bacterial attachment to surfaces. Its normal function is to maintain the cell wall by hydrolyzing the peptidoglycan (PGN) (18, 19, 20, 21). Autolysin interacts with PGN-attached wall teichoic acid and membrane-attached lipoteichoic acid (LTA), and the differential distribution of LTA and wall teichoic acid directs autolysin to the cell division site (22, 23). In biofilms, AtlE mediates the initial bacterial attachment to polystyrene surfaces and extracellular matrix proteins (24, 25).

AtlE is translated as a single polypeptide containing an N-terminal propeptide, an amidase (Ami) catalytic domain, three structural GW repeats (R1ab, R2ab, R3ab), and a glucosaminidase catalytic domain (Glc, Fig. 1*A*) (21, 26, 27). Proteolytic processing of AtlE results in an Ami fragment (Ami-R1ab-R2ab) and a glucosaminidase fragment (R3ab-Glc). The R1ab and R2ab repeats tether Ami noncovalently to the cell wall (28, 29), whereas the R3ab repeat performs a similar function by mediating the tethering and targeting of Glc domain to the septum during cell division (30). Recently, Nega *et al.* have shown that deletion of Ami or Glc domains in *Staphylococcus* leads to abnormal cell clustering and asymmetric cell division (30). The Ami repeats mediate biofilm formation and have been shown to interact with human extracellular matrix proteins (31, 32). The R2ab repeats also facilitate attachment to polystyrene surfaces (24), and we recently showed that treating polystyrene surfaces with R2ab reduces staphylococcal biofilm formation relative to treatment with serum albumin (33), and R2ab-functionalized nanoparticles can drive particle localization to biofilms (34). The structural properties of R2ab involved in attachment, however, remain unclear. Protein interaction with surfaces involves numerous events, which may be transient or weak (35). Moreover, structural analysis using X-ray crystallography and cryo-electron microscopy is challenging because of the lack of regular protein orientations on a surface (36). Understanding how the Ami R2ab repeat mediates surface attachment could lead to new approaches for reducing biofilm related infections.

**Figure 1:**
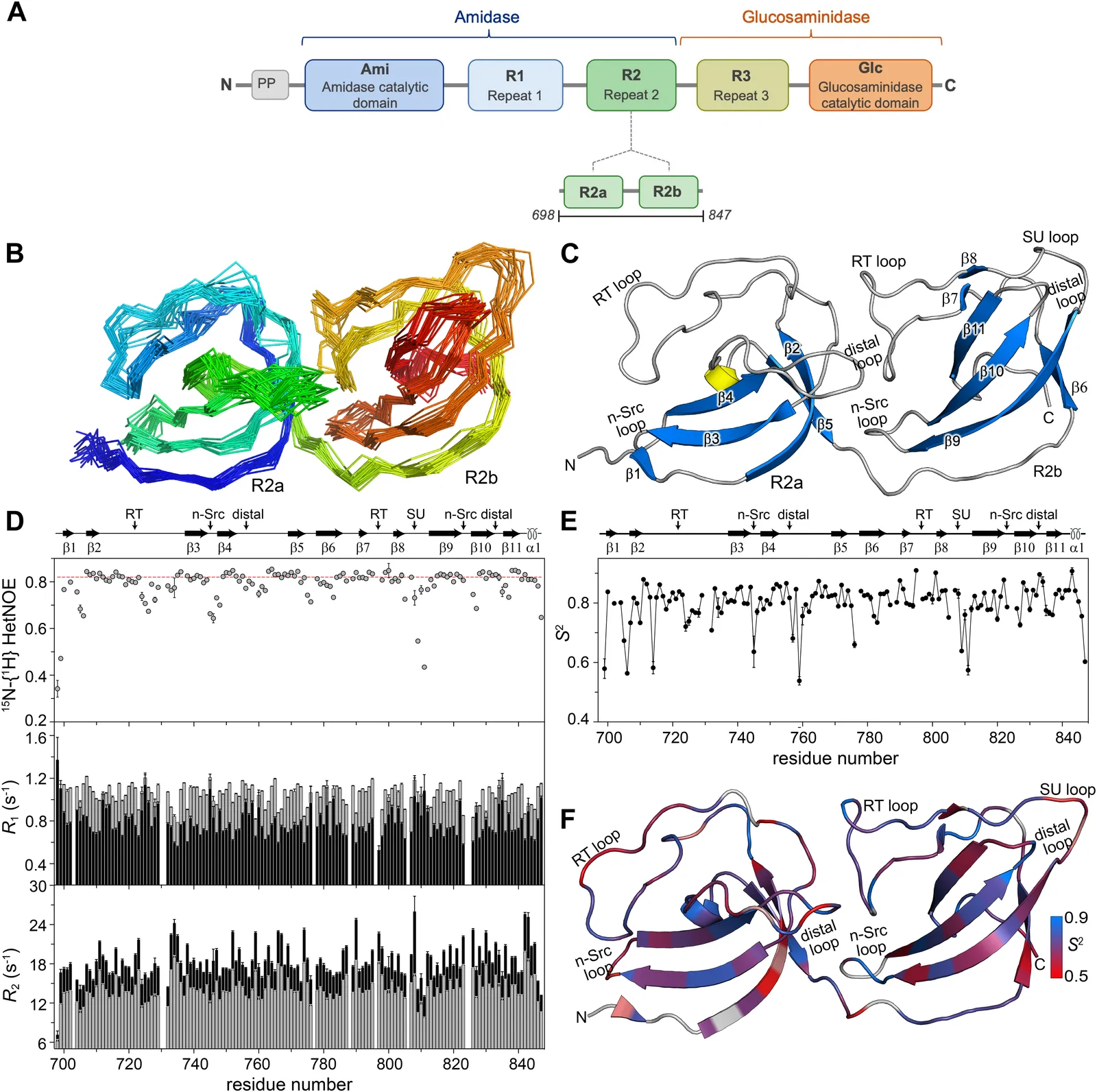
**Solution structure and backbone dynamics of R2ab.***A*, the domain organization of AtlE precursor protein. AtlE consists of propeptide, amidase (Ami-R1ab-R2ab), and glucosaminidase (R3ab-Glc catalytic domain) domains. *B*, superimposition of backbone traces from a final ensemble of 20 structures with the lowest total energy from final CNS v1.3 refinement. *C*, ribbon diagram of a representative structure of R2ab showing subunits *a and b*, with a half-open β-barrel formed by six β-strands (PDB ID: 7KWI). *D*, ^15^N relaxation parameters. ^15^N-{^1^H} hetNOE *(top)*; *R*_1_*(middle)*; *R*_2_*(bottom)* values of R2ab obtained at 25 °C at 600 *(gray circle or bar)* and 900 MHz *(black bar)* spectrometer. The theoretical maximum NOE at 600 MHz (0.82) is indicated as a *red dashed line*. The *error bar* in the hetNOE experiment represents the uncertainty based on experimental noise, while the *R*_1_ and *R*_2_*error bars* indicate the *fitting error*. *E*, the generalized order parameter (*S*^2^) obtained from the optimized model-free analysis using the Relax program is shown as a function of residue number. *F*, the generalized order parameter is color-coded from *blue* (*S*^2^ = 0.9) to red (*S*^2^ = 0.5). Excluded residues that are not included in the model-free analysis are colored *gray*. hetNOE, heteronuclear NOE.

Given R2abʼs role in cell wall restructuring and its function as an adhesin to abiotic surfaces in *S. epidermidis* biofilms, we sought to understand the mechanism of attachment using solution nuclear magnetic resonance (NMR) spectroscopy and calorimetry. We have determined the solution structure of R2ab and used NMR relaxation to identify molecular motions that facilitate cell wall binding. Isothermal titration calorimetry (ITC) is used to identify two thermodynamically distinct binding sites for LTA. NMR titrations identified the primary LTA-binding site on R2ab. For the first time, the PGN binding site is also identified, and NMR data are used to generate a model for cell wall binding. The structures reveal that both subdomains are involved in binding, but little overall structural change occurs. Finally, we develop a new approach to monitor polystyrene surface binding in R2ab. Polystyrene nanoparticles (PSNPs) are paramagnetically labeled at low levels, and interaction sites are monitored in R2ab at the residue level. These data are used to identify a binding interface on R2ab, from which a model of how R2ab mediates bacterial attachment to polystyrene surfaces was constructed. To confirm this model, we designed R2ab variants and showed that they were significantly less effective at inhibiting biofilm growth on polystyrene than WT R2ab. Together, our results establish that strong interactions between R2ab and cell wall components, along with a well-defined polystyrene binding surface, can result in a structurally stable initial attachment complex during *S. epidermidis* attachment. Moreover, such a complex could be targeted to hinder biofilm formation.

## Results

### R2ab solution structure: significant loop motions, but one single cooperative unit

The ^1^H–^15^N heteronuclear single-quantum coherence (HSQC) spectrum of R2ab exhibits good dispersion, with 141 out of 149 backbone amides assigned (Fig. S1 and Table S1). The solution structure ensemble of 20 structures (PDB ID 7KWI) is tightly clustered, with an RMSD of 1.0 and 1.5 Å for ordered backbone and heavy atoms, respectively (Fig. 1, *B* and *C*, and Table S2). The structure of R2ab consists of two half-open β-barrel subunits, each consisting of at least five β-strands. Both R2a and R2b subunits have several loops common to SH3b domains: RT, SU, distal, and n-Src loops. The long, flexible RT loops connect strands β2-β3 (residues 710–735) and β7-β8 (787–810), respectively. Both the RT loops and the distal loops, between strands β4-β5 (V752–N760) and β10-β11 (V833–K836), are longer in the R2a subdomain. The central β-sheet of each subunit has a tryptophan residue (W763 in R2a and W839 in R2b) preceded by a glycine (GW motif) (Fig. S2); this GW motif is a conserved feature of autolysin repeats and plays an important role in forming a ligand-binding pocket. The C-terminus of R2ab is characterized by a short turn-helix (K842–L845), and the presence of strong NOEs in this region indicates restricted motion of the C-terminal residues. The interface between the subunits is stable, as side-chain NOEs are observed between residues in β5 of R2a and in the RT and n-Src loops of the R2b subunit.

The solution structure compares favorably with a previously determined crystal structure of R2ab (PDB 4EPC) (29). The global superposition RMSD is 1.9 Å (37), and the secondary structures are similar for both structures. However, the RT and distal loop of the R2a subunit, and the SU loop of the R2b subunit show higher RMS deviations, suggesting greater flexibility in these loops (Fig. S2). The positioning of the RT loop, along with the residues of β1 and β3 in the R2a subunit, forms a putative ligand-binding site, while a second ligand-binding pocket is present closer to the C-terminal part of the R2b subunit (29). While Zoll *et al.* were not able to determine an experimental structure of R2ab bound to LTA or PGN, the structural differences in these loop regions suggest that the RT loop motions in the R2a and R2b subunits play an important role in ligand binding. To understand these motions, we next determined the backbone amide relaxation rates.

The ^15^N relaxation rates and the steady-state ^15^N-{^1^H} heteronuclear NOE (hetNOE) highlight the significant motions that occur in R2abʼs loop regions (Fig. 1, *D*–*F*). Relaxation rates were collected at 600 MHz and 900 MHz fields, and model-free analysis was used to characterize protein motions (38, 39, 40). As anticipated, increasing magnetic field strength led to a decrease in the R_1_ rate and an increase in the R_2_ rate (Table S3). This caused the R_2_/R_1_ ratio to nearly double at higher fields, indicating an apparent rotational correlation time of about 11.4 ns. Model-free analysis of these measurements (38, 41) produced an average rotational correlation time (τ_m_) of 11.1 ns and a mean generalized order parameter (*S*^2^) of 0.80 ± 0.01 for the residues analyzed (Table S3). Reduced hetNOE and generalized order parameter (*S*^*2*^) values reflect increased motions in the R2a RT and n-Src loops; these motions are not observed in the RT loop of the R2b subunit (Fig. 1*D top*). Overall, the R2b subunit is less dynamic with one exception: The SU loop in R2b exhibits lower order parameters (*S*^2^, Fig. 1*E*) and hetNOE values. Residues in this SU loop were fit to a two-site chemical exchange model; the average relaxation contributions (*R*_*ex*_) to chemical exchange for the SU loop are in the 2 to 4 s^−1^ range. This corresponds to motions in the SU loop on the μs-ms timescale that may be important for substrate binding (Fig. S3*A*). On the other hand, the RT and n-Src loops in R2b directly interact with R2a, which may account for the decreased motions of these loops in the R2b domain relative to R2a. Motions in the N-terminus and C-terminus are also starkly different between the subunits: N-terminal hetNOE values drop to 0.19 and remain low until the start of β2 (residue N703); in contrast, the C-terminal residues, comprising β11 and the final helical turn, exhibit high hetNOE and *S*^2^ values. A final question is whether the R2a and R2b are rotationally rigid or tumble independently. Prior work suggested that the R2a and R2b domains were rigid: small-angle X-ray scattering data fit well with the crystal structure, and lower b-factors were observed in the linker region (T773–S776) (29). Our data are consistent with this finding; the average hetNOE value of the linker is 0.76, indicating that it is relatively rigid. In addition, relaxation rates are consistent with coupled rotational diffusion of both subunits. R_1_ and R_2_ rates fit well to an anisotropic diffusion model, and despite significant loop motions, both domains appear to rotate as a single unit with an effective rotational correlation time (*τ*_c_) of 11.3 ns (Fig. S3*B*), which is reasonable for a 16.5 kDa protein.

Although the subunits of R2ab are rotationally rigid, it is possible that they fold independently. To test this, we performed fluorescence chemical denaturation and differential scanning calorimetry experiments. A comparison of Van’t Hoff and calorimetric enthalpy suggests that both domains fold as a single cooperative unit. The calorimetric melting temperature (T_m_) and enthalpy (Δ*H*_cal_) were estimated to be 50.7 °C and 92.8 ± 0.9 kcal/mol, respectively (42, 43) (Fig. S4*A*). This thermal transition was reversible, with no aggregation observed during denaturation. Chemical denaturation experiments (Fig. S4, *B* and *C*) were used to determine a Van’t Hoff enthalpy (Δ*H*_VH_) of 70 ± 8 kcal/mol, in moderate agreement. While it is possible that an intermediate state is present during unfolding, unfolding transitions show no evidence of multi-state behavior. Moreover, the denaturant *m*-value is consistent with cooperative unfolding (44). This value was determined to be 4.4 ± 0.4 kcal mol^−1^ M^−1^, corresponding to a change in solvent-accessible surface area of 16,000 Å^2^. Using our structure, we estimate solvent-accessible surface area to be between 8400 and 17,400 Å^2^ (45, 46, 47), which is consistent with both subunits unfolding cooperatively.

### Interactions with cell wall components (LTA and PGN)

Previous studies have shown that bacterial SH3 domains can interact with LTA and PGN (48, 49). Biswas *et al.* first reported that AtlE repeat domains can directly interact with PGN and LTA, in fact, the presence of R1ab and R2ab repeat domains has been shown to enhance the Ami PGN hydrolase activity (26). Furthermore, preincubating LTA with Ami-R1ab-R2ab decreases its PGN-hydrolyzing activity in a dose-dependent manner, suggesting that LTA may have greater specificity for the R1ab-R2ab repeats (29). Therefore, we used ITC, solution NMR, and molecular docking to characterize the interactions between R2ab repeats and LTA and PGN.

Purified *Staphylococcus aureus* LTA was first titrated with the R2ab, and the ITC thermogram was collected. Although the precise concentration of LTA and, therefore, the molar ratio of LTA to R2ab in the ITC experiment could not be accurately ascertained due to the heterogeneity of the commercially available purified LTA, the ITC data nonetheless inform the thermodynamic binding parameters of the R2ab-LTA interaction. The ITC binding curve fitting indicates that the isotherm can be described by the two-site binding model, consistent with the previous study, in which mutations in the R2a (A742L) and R2b (H793R, S841E) subunits resulted in the loss of LTA binding (29). The comparison of the two binding sites reveals differences in the thermodynamic binding parameters of the LTA interaction onto the two binding sites of R2ab. It is clear from the isotherm that the interaction of LTA with the first binding site on R2ab is characterized by higher affinity (*K*_d_ 0.15 ± 0.1 μM) with exothermic reaction and favorable binding enthalpy. In contrast, the interaction of the second LTA site is characterized by a six times weaker affinity (∼ *K*_d_ 1.0 ± 0.2 μM) with an endothermic reaction (Fig. 2*A*). Although it is clear that R2ab has two LTA-binding sites, likely one on each subunit, it is not clear which subunit has a higher affinity for LTA. Therefore, to further characterize this interaction, LTA (60 and 120 μg) was mixed with 100 μM R2ab, and a 2D ^1^H–^15^N HSQC experiment was collected. The resulting data, when compared to the reference sample (without LTA), show a concentration-dependent loss of R2ab amide peak intensity in the presence of LTA, indicating direct interaction of LTA with R2ab (Figs. 2*B*, *C*, and S5*A*). Although no chemical shift perturbations (CSPs) were observed, the loss of amide peak intensity clearly highlights a contiguous region in the R2a subunit that experiences larger than average intensity loss upon LTA binding in comparison to the other regions in the protein. This region in the R2a subunit is formed by the residues of the RT loop (G716, L717, Y718, T719, T720, Y722, D723), β3 (K740, L744), and GW motif (G762 and W763) in the R2a subunit (Figs. 2*C* and S5*B*). This region in the R2a subunit is similar to that identified previously; however, the second LTA binding site, which was shown to lie on the posterior face of the R2b subunit (29), could not be identified in the same NMR experiment. The NMR data suggest that R2a could be the high-affinity LTA-binding site of R2ab. The addition of excess LTA (500 μg) in similar experimental conditions resulted in the complete loss of the R2ab amide signal dispersion, indicating the formation of soluble R2ab and LTA aggregate (Fig. S5*A*). Further, docking of LTA with R2ab was performed to gain better insight into its binding mode. An LTA model was generated based on the study by Morath *et al.* (50), and conformation optimization and energy minimization were performed. A grid box was generated for the docking of LTA on the R2a subunit based on structural information obtained from NMR. The docked model of LTA onto the R2a subunit shows that mainly the teichoic acid part of the LTA interacts with the R2a subunit, in which residues of the RT loop make important hydrophobic interactions, and the residues T719, T720, T724, K740, and N760 make hydrogen bonds with the teichoic acid (Figs. 2*D* and S5*C*). Moreover, the glycerophosphate chain of the LTA, which functions as an anchor, does not interact significantly with the R2a subunit.

**Figure 2:**
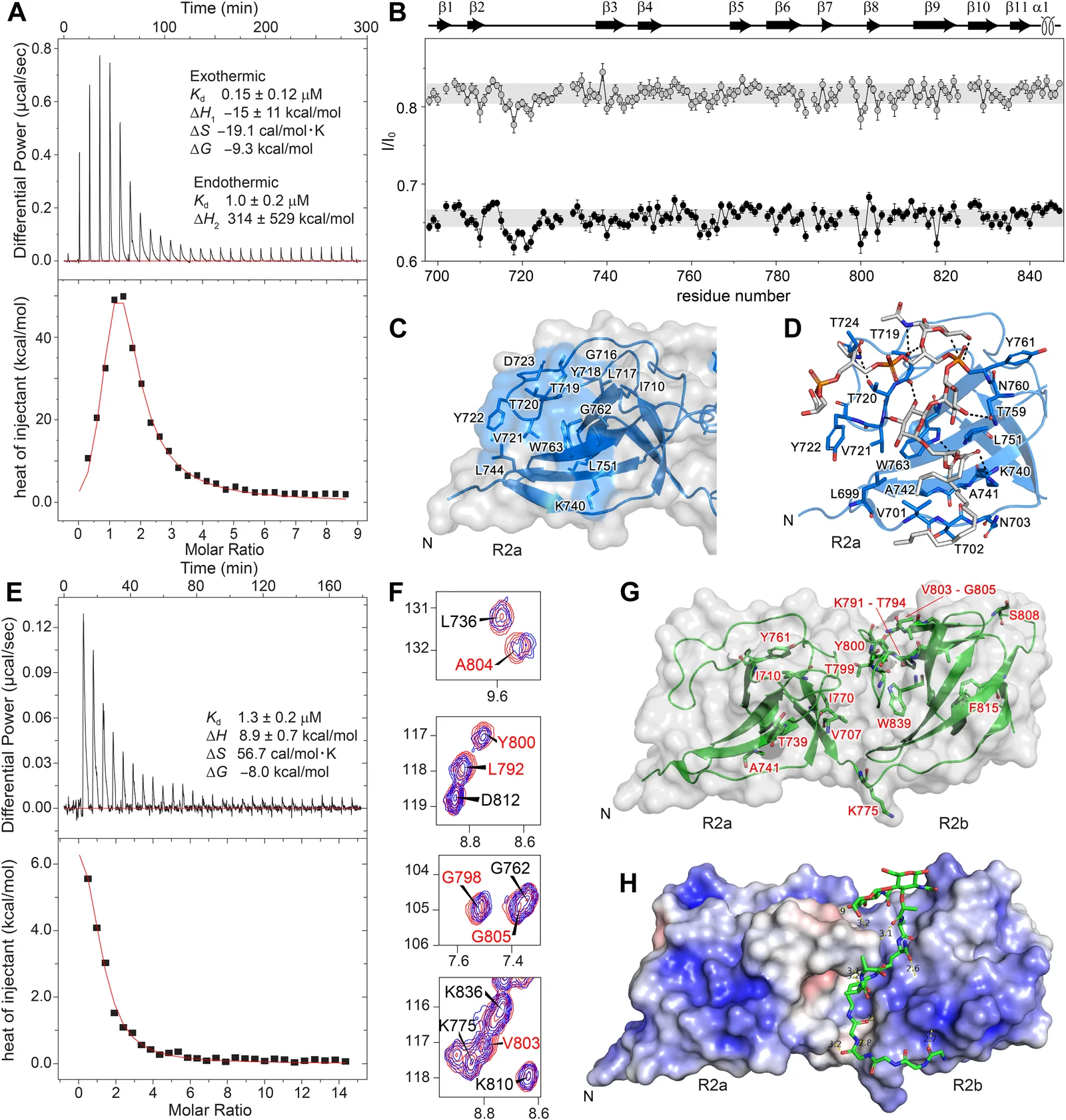
**Interaction of LTA and PGN with the R2ab.***A*, thermodynamic parameters indicate the presence of two LTA-binding sites on R2ab. The raw thermogram *(top panel)* and the binding isotherm from the integrated thermogram fit using a two-binding-site model in Origin v7 *(bottom panel)* are shown. *B*, peak intensity ratio (I/I_0_) of R2ab signals (100 μM) in the absence and presence of 60 *(gray circles)* or 120 μg *(black circles)* LTA in a sample volume of 500 μl. The highlighted *gray* region represents the mean plus two standard deviations: 0.82 ± 0.02 and 0.66 ± 0.02 at 60 and 120 μg, respectively. *C*, the prominent LTA-binding site on the R2a subunit, as indicated by the peak intensity ratio in *(B)*, is highlighted in *blue* and labeled on the R2a surface. *D*, the docked pose of the LTA onto the R2a structure. The docking was performed using the CCDC-GOLD flexible docking program, and poses were ranked and selected based on the Chemscore function. Residues that participate in either electrostatic or hydrophobic interactions are labeled. *E*, ITC thermogram and binding isotherm from the integrated thermogram for PGN binding fit using a one-binding-site model in Origin v7 *(bottom panel)* are shown. *F*, selected regions of the ^1^H–^15^N HSQC of R2ab showing CSP in the absence *(red)* or presence *(blue)* of PGN. *G*, residues showing CSPs upon PGN binding are mapped onto the structure of the R2ab. *H*, the docked pose of PGN onto the R2ab structure. The docking was performed using the CCDC-GOLD flexible docking program, and docking poses were ranked and selected based on the Chemscore function. The surface representation highlights the ligand cavity between the R2a and R2b subunits. The Adaptive Poisson-Boltzmann Solver electrostatic surface potential of the R2ab, determined using the Adaptive Poisson-Boltzmann Solver algorithm and the PDB2PQR program (80), highlights the positively charged residues lining the ligand-binding cavity. LTA, lipoteichoic acid; CSP, chemical shift perturbation.

To determine the interaction between PGN and the R2ab domain, purified *S. aureus* PGN was titrated into R2ab. The ITC binding curve indicates an endothermic reaction with micromolar affinity (*K*_d_ 1.3 ± 0.2 μM) of PGN for R2ab (Fig. 2*E*). However, the ITC experiment indicates that the PGN-R2ab binding isotherm could be fit with the one-site binding model, in contrast to the two-binding-site model for the LTA and R2ab interaction. To further characterize the binding of PGN with R2ab, a 2D ^1^H–^15^N HSQC spectrum of R2ab was collected in the presence and absence of PGN (Fig. S6*A*). The comparison of the HSQC spectra shows weaker CSPs for specific residues; however, further CSP mapping could not be performed due to visible aggregation in the sample upon adding a higher amount of PGN, which resulted in the complete loss of amide signal intensity for R2ab residues. The aggregation of R2ab induced by the addition of excess PGN differed from that of LTA; while excess LTA caused soluble aggregation of R2ab, the addition of excess PGN to R2ab resulted in a visible aggregate. Mapping of residues experiencing CSP upon PGN binding on the structure of R2ab indicates residues of R2a (V707, T739, Y755, Y761, and I770) and R2b subunit (K775, L792, T794, Y800, S808, L829, and W839) form a ligand-binding cavity (Figs. 2*F* and S6*B*). Similar to LTA, PGN was also docked onto R2ab, and a docking grid was generated based on the CSPs observed in the NMR experiment. A complete PGN subunit comprising N-acetylglucosamine, N-acetylmuramic acid, the PGN peptide stem (l-Ala, d-Gln, l-Lys, d-Ala, d-Ala), and the pentaglycine cross-bridge was generated, its conformation optimized, and its energy minimized. The docked model of PGN binding to R2ab indicates that the ligand-binding site, lined by many polar and charged residues (S705, N756, K775, K779, and K819), is suited to accommodate PGN, as these charged residues can form hydrogen bonds with the carbonyl of the PGN peptide stem (Fig. 2, *G* and *H*). Comparison of the PGN pentaglycine bound *S. simulans* lysostaphin SH3b domain (48) with the R2ab also suggests the propensity of PGN to bind a similar site on the R2ab. Moreover, our results suggest that LTA and PGN do not bind to the same site on the R2a subunit and that, overall, LTA shows a greater propensity to bind R2ab than PGN.

### Interaction and orientation of R2ab on a polystyrene surface

AtlE is directly implicated in *S. epidermidis* biofilm formation, because a significant decrease in the bacterial recovery was observed from the explanted catheters when inoculated with the AtlE-negative *S. epidermidis* (51). Experiments by Heilmann *et al.* suggest that the amidase containing fragment (Ami-R1ab-R2ab) is responsible for AtlE’s attachment to the polystyrene surfaces (24). Previously, our group has studied the thermodynamics of interaction between R2ab repeats and various polystyrene nanoparticles (anionic, carboxylate-functionalized, and non-functionalized) (33, 52, 53). Here, we have characterized the physicochemical properties and determined the orientation of R2ab upon adsorption onto the carboxylate polystyrene nanoparticles (PSNP) using NMR spectroscopy and ITC.

To characterize the interaction and orientation of the R2ab with PSNP, a paramagnetic relaxation enhancement experiment was used. The carboxyl functional group of PSNP was modified with a paramagnetic spin-label 4-amino-2,2,6,6-tetramethylpiperidine-1-oxyl (4-amino-TEMPO) using 1-ethyl-3-(3-dimethylaminopropyl) carbodiimide (EDC) and N-hydroxysulfosuccinimide (sulfo-NHS) crosslinking chemistry (Fig. 3*A*). The spin-label modification of PSNP increased the PSNP diameter from 56 nm to 75 nm, but the overall surface charge remained similar (*ζ* −32 mV for PSNP and −35 mV for PSNP@TEMPO) (Fig. S7). Varying amounts of PSNP@TEMPO were added to a fixed concentration of ^15^N R2ab, and 2D ^1^H–^15^N HSQC were collected. A reduction in the R2ab amide proton intensity was observed in the presence of PSNP@TEMPO; importantly, only specific residues/regions experienced the PRE effect (Fig. S8). To ascertain that the PRE effect is due to the spin-label, the free radical was quenched by adding ascorbic acid to the duplicate samples. An intensity ratio of amide protons in the absence and presence of a free radical (I_para_/I_dia_) clearly shows distinct residues/regions of R2ab with diminishing intensity as the PSNP@TEMPO concentration increases. The analysis of the PRE effect on R2ab suggests that residues of the R2ab loop region are more affected than buried, static residues (Fig. 3*B*). Moreover, mapping the residues with a significant PRE effect onto the R2ab structure reveals a putative polystyrene binding region. A few scattered low-intensity residues are observed on the front-facing R2a subunit (S705, A712, V721, and Y722) and front-facing R2b subunit (S776 and S837). However, most of the residues (V790, K791, W797, Y800, G809, A826, T827, S841, Y843, and A847) with low I_para_/I_dia_ were located on the back-facing C-terminal face of the R2b subunit, forming a large contiguous region (Fig. 3*C*). Although the PRE effect on the residues of the R2a subunit is intriguing, it could be indicative of more than one binding mode of protein to a nanoparticle, which often involves initial binding and rearrangement events as more protein molecules compete for the limited binding sites on a nanoparticle. The structural and biophysical basis of R2ab interaction with the PSNP surface remains an area of active investigation; nonetheless, an overwhelming grouping of residues on the R2b subunit suggests that R2ab might interact with the PSNP surface *via* the C-terminal face of the R2b subunit.

**Figure 3:**
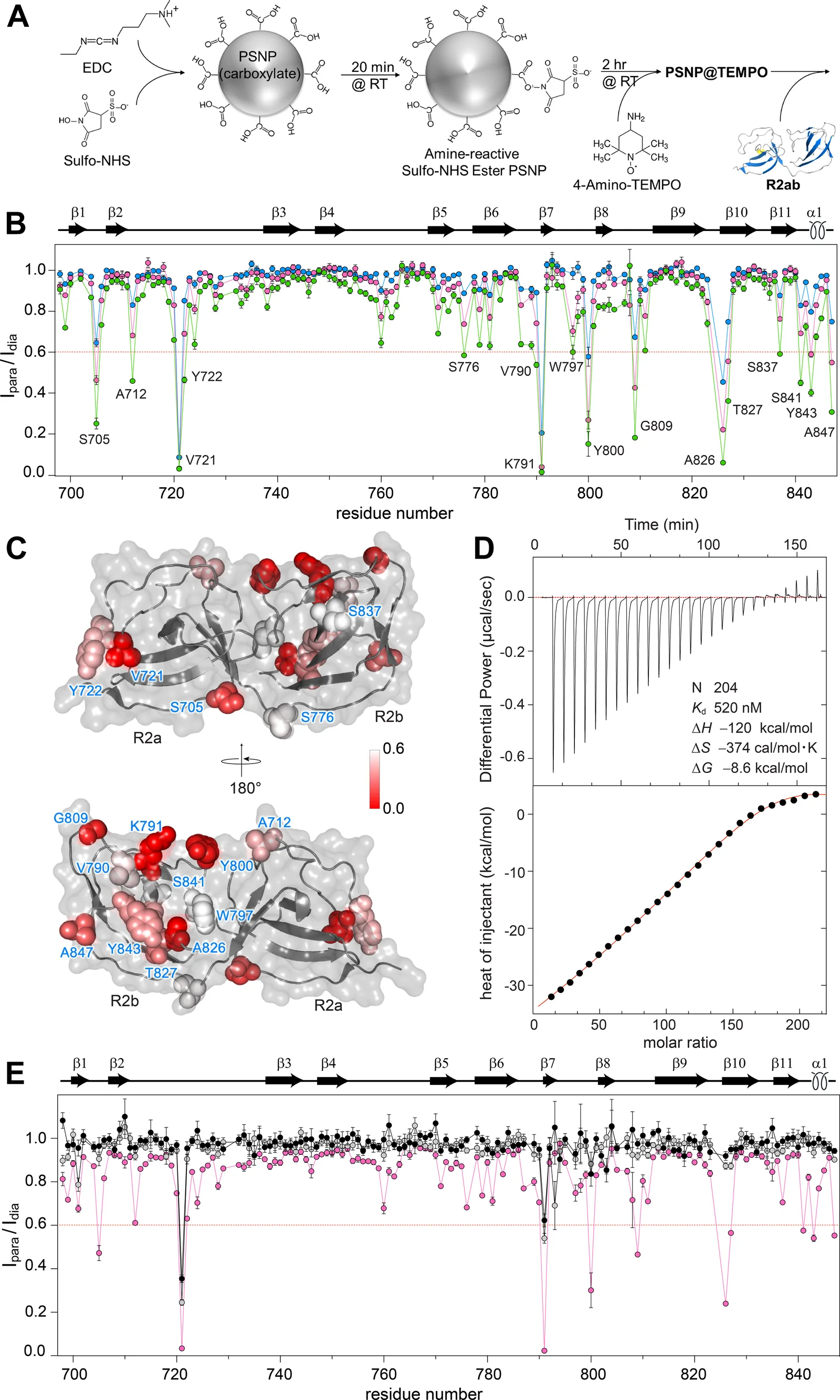
**The R2ab and carboxylate PSNP interaction.***A*, spin-labeling scheme of carboxylate-PSNP is shown. *B*, the intensity ratio (I_para_/I_dia_) of the 100 μM R2ab in the presence of spin-labeled PSNP@TEMPO at 10 nM (blue), 20 nM (magenta)and 40 nM (*green*), *versus* free radical quenched PSNP@TEMPO. The *red dashed line* represents the cut-off for the significant PRE effect. *C*, the surface representation of the R2ab structure. The highlighted residues are represented as spheres and shaded according to the I_para_/I_dia_ for the highest PSNP@TEMPO concentration. The I_para_/I_dia_ values are color-coded from *white* (0.6) to *red* (0.0). *D*, baseline-corrected raw thermogram and binding isotherm from the integrated thermogram fit using the CHASM (78) two-binding site model are shown. Thermodynamic parameters reveal multiple R2ab molecules bind to PSNP (50 nm) with submicromolar affinity. *E*, the intensity ratio (I_para_/I_dia_) of the R2ab (100 μM) in the presence of 20 nM PSNP@TEMPO *versus* free radical quenched PSNP@TEMPO *(magenta circles)*. The *red dashed line* represents the cut-off for the significant PRE effect. Increasing ionic strength to 100 *(gray circles)* and 200 mM *(black circles)* NaCl progressively diminishes signal attenuation, indicating reduced interaction between R2ab and PSNP@TEMPO. PSNP, polystyrene nanoparticle.

To further characterize the R2ab interaction with PSNP, R2ab was titrated into PSNP, which resulted in an exothermic reaction with approximately 200 R2ab molecules interacting with one PSNP (Fig. 3*D*). However, the ITC binding isotherm could not be fit with one site binding model as the isotherm showed an endothermic reaction after saturation of the binding sites, which perhaps indicates a weak interaction of R2ab on top of adsorbed R2ab monolayer (54). This multilayer phenomenon is not uncommon and has been shown for other proteins (52, 55). Fitting the binding isotherm to the two-site binding model suggests a sub-micromolar affinity (*K*_d_ = 520 nM) of R2ab for PSNP. Moreover, the adsorption of R2ab onto PSNP is driven by a favorable enthalpic contribution, consistent with electrostatic interactions contributing to its adsorption to carboxylate-PSNP.

To evaluate the role of electrostatics in the interaction between R2ab and PSNP@TEMPO, NaCl was added at concentrations of 100 and 200 mM to samples containing 100 μM R2ab and 20 nM PSNP@TEMPO. Increasing salt concentrations attenuated PSNP@TEMPO binding, as evidenced by increased I_para_/I_dia_ ratios (Fig. 3*E*). These results indicate a weakened interaction between R2ab and PSNP@TEMPO at higher salt concentrations, supporting the contribution of electrostatic forces to the adsorption process.

### Precoating of R2ab on polystyrene disrupts biofilm growth on polystyrene

The spin-label experiments with PSNPs indicated that specific regions of R2ab are likely involved in surface adsorption. Therefore, specific point mutations (Y722A, S776A, K779A, Y800A, Y843A, and Y844A) in R2ab were made, and the effect of these mutations on the polystyrene surface adsorption and subsequent *S. epidermidis* biofilm growth on the polystyrene surface was ascertained. We chose to replace serine (S776), lysine (K779), and tyrosine (Y722, Y800, Y843, Y844) residues with alanine to reduce surface charge and hydrophobicity. Residue positions were selected based on the region identified above (Fig. 3*C*).

Previously, our group has shown that pretreating a polystyrene microtiter plate with the R2ab repeat significantly reduces *S. epidermidis* biofilm formation without affecting bacterial growth, in contrast to pretreatment with bovine serum albumin (33). Taking advantage of *S. epidermidis* biofilm growth on the protein-pretreated polystyrene surface, a similar assay was carried out. As observed previously, pretreatment of polystyrene microtiter wells with WT R2ab results in significantly reduced biofilm in comparison to the untreated or the BSA-treated wells. Surprisingly, tyrosine mutants (Y722A, Y800A, Y843A, and Y844A) did not show enhanced biofilm formation, indicating that these mutants adhered to polystyrene similarly to WT R2ab. In contrast, both S776A and K779A mutants showed significantly higher *S. epidermidis* biofilm in comparison to either WT R2ab or tyrosine-mutated R2ab. This observation is consistent with reduced R2ab adsorption to the polystyrene surface, allowing *S. epidermidis* to form biofilm more readily (Figs. 4*A* and S9).

**Figure 4:**
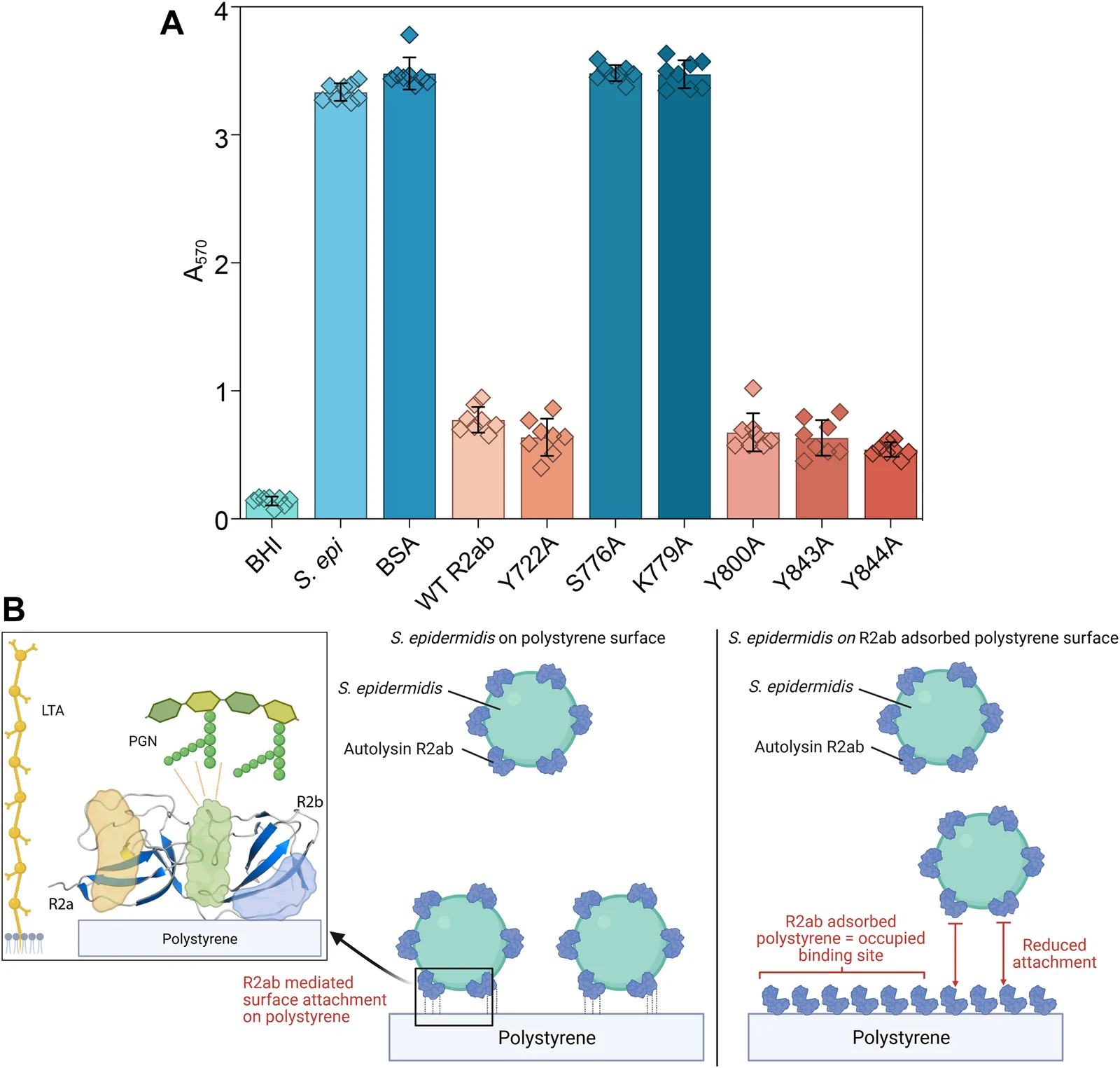
***S. epidermidis* biofilm on polystyrene surface in the presence of R2ab-wt and R2ab mutants.***A*, the effect of the R2ab-wt and R2ab mutants’ preincubation on the *S. epidermidis* biofilm formation. Wells were precoated with either BSA or the WT R2ab or R2ab mutants. Wells were then washed to remove unbound protein, followed by the addition of *S. epidermidis* in brain heart infusion medium, and biofilm formation was quantified by *crystal violet* staining. *Error bars* represent the standard error of the mean for N = 8 samples. BHI was used as a negative control; *S. epidermidis* and BSA were used as positive controls. *B*, scheme illustrating R2ab mediates bacterial attachment to bare polystyrene. In the *middle*, R2ab on the bacterial surface readily supports bacterial adhesion to polystyrene through direct R2ab-surface interactions, indicated by *dotted lines*. On the *right*, precoating the polystyrene surface with purified R2ab, followed by washing to remove unbound protein, occupies available binding sites, thereby reducing subsequent bacterial attachment. The inset on the *left* depicts a model of R2ab interactions with bacterial cell wall components, LTA and PGN, and polystyrene surface. The figure was created using BioRender.com. LTA, lipoteichoic acid; PGN, peptidoglycan.

These results highlight the crucial role of polar and charged residues (S776 and K779) in polystyrene adsorption. Moreover, both residues lie near the border of the proposed binding region on the C-terminal side of the R2b subdomain, highlighting the relevance of this region. Notably, these two residues sit at the border rather than the center of the proposed interface, suggesting they may act as specific anchoring contacts rather than contributing to a broad adsorption footprint. The fact that tyrosine mutations did not significantly affect biofilm formation could be due to several factors. Firstly, the tyrosine-to-alanine mutations reduce steric bulk. Reducing steric bulk at a single residue may allow nearby residues to bind more easily, compensating for the mutation. Such an effect is observed for glycine and alanine variants on gold nanoparticles (56). Secondly, partial protein unfolding may complicate the structural interpretation of PRE-derived binding interfaces. Previously, we proposed a model whereby proteins can partially unfold when adsorbed onto polystyrene (52). While PRE measurements with polystyrene measure the early association complex, where R2ab approaches the surface, this biofilm assay covers a much longer timescale, and we cannot rule out larger structural changes that occur after adsorption. Nevertheless, the fact that S776 and K779 can alter adsorption so dramatically suggests that R2ab has preferred contacts when adsorbed to polystyrene surfaces. Future work with more variants is needed to establish a more complete picture of the R2ab-polystyrene interface.

To explain the observed binding of LTA, PGN, and polystyrene by R2ab, we propose a model in which the R2ab domain contains two distinct ligand-binding sites: the R2a subunit harbors a prominent LTA-binding site, whereas the groove formed between the R2a and R2b subunits constitutes a PGN-binding site. In addition, residues within the R2b subdomain are proposed to mediate the initial interaction with the polystyrene surface (Fig. 4*B*). In this model, the R2ab domain anchored *via* the LTA and/or PGN on the bacterial surface functions as an adhesin that promotes attachment to polystyrene. Upon contact with the abiotic surface, R2ab adsorbs onto polystyrene while maintaining its engagement with LTA and/or PGN molecules displayed on the bacterial cell envelope, thereby stabilizing bacterial adhesion to the polystyrene surface. This mechanism provides a structural basis for the direct involvement of R2ab in primary attachment.

Consistent with this model, precoating the polystyrene surface with purified R2ab reduced bacterial attachment. We propose that adsorbed R2ab molecules occupy the surface-binding sites normally recognized during bacterial adhesion. As a result, incoming bacteria encounter an R2ab-coated surface where the available adhesion sites have already been saturated, preventing productive interactions between the bacterial surface-associated R2ab and the polystyrene substrate (Fig. 4*B*). Together, these findings support a mechanism in which R2ab acts as a bifunctional cell wall-binding protein that not only recognizes Gram-positive cell wall polymers but also directly mediates *S. epidermidis* adhesion to abiotic polystyrene surfaces. Surface immobilization of purified R2ab effectively masks the binding sites required for this interaction, thereby inhibiting the initial attachment step that precedes biofilm development.

## Discussion

Protein-mediated initial attachment to abiotic surfaces is a critical first step in *S. epidermidis* biofilm formation on medical devices. The R2ab domain of the staphylococcal autolysin AtlE has been directly implicated in this process (24), but the structural basis for how R2ab engages cell wall components and polystyrene surfaces, potentially simultaneously, remains undefined. Using solution NMR spectroscopy and calorimetry, along with molecular docking, paramagnetically labeled polystyrene nanoparticles, and biofilm assays, we determined the solution structure and backbone dynamics of R2ab and characterized its interactions with LTA, PGN, and polystyrene nanoparticles at residue resolution. These results show that R2ab is a structurally stable SH3b-like repeats containing distinct yet functionally connected surfaces for cell wall ligand binding and polystyrene surface interaction.

The solution structure of R2ab confirms two compact half-open β-barrel subunits, R2a and R2b, that resemble bacterial SH3b-like domains. Bacterial SH3b domains are structurally related to eukaryotic SH3 domains, but they are most commonly found in bacterial and phage cell wall hydrolases, where they function as cell wall-targeting modules that direct catalytic domains to the bacterial cell wall (48, 57). In R2ab, each repeat contains loop regions that are characteristic of SH3/SH3b-like folds, including RT, n-Src, distal, and SU loops. These loops are important because ligand recognition in SH3b-like domains is often mediated by surface grooves or cavities formed near these loops and β-strand regions. Within each subunit, the conserved GW motif (W763 in R2a, W839 in R2b) contributes to the central ligand-binding face (29). For example, in lysostaphin, the SH3b domain recognizes the staphylococcal peptidoglycan pentaglycine bridge *via* a groove formed near the β1 and β2 strands (48). The ^15^N relaxation measurements reveal an asymmetry between subunits: the RT and n-Src loops of R2a are more mobile on the fast (ps–ns) NMR timescale, while the corresponding R2b loops are held rigid by contacts at the subunit interface. Despite these local loop motions, both subunits behave as a single cooperative unit, as backbone dynamics and relaxation analysis indicate that the two repeats tumble together as one unit. Furthermore, thermal and chemical denaturation support this model, as R2ab unfolds cooperatively rather than as two independent subdomains. Thus, R2ab appears to use local loop motions for ligand recognition while maintaining a globally stable scaffold.

The interaction of R2ab with LTA and PGN supports the idea that the tandem SH3b-like fold provides a multi-ligand cell wall recognition surface. Previous work has shown that AtlE repeat domains interact with both LTA and PGN and that these interactions are important for positioning the catalytic domain near the cell wall (26, 29). Our ITC data show that R2ab binds to two LTA ligands, with thermodynamically distinct binding events. The higher-affinity site is exothermic, whereas the second binding event is weaker and endothermic. Although the heterogeneity of purified LTA prevents exact determination of ligand stoichiometry, these data are consistent with the presence of more than one LTA interaction surface in the tandem repeat. This two-site profile is consistent with Zoll *et al.*'s mutagenesis, which showed that point mutations in both R2a (A742L) and R2b (H793R, S841E) independently abolished LTA binding (29), supporting the view that each subunit contributes one binding event. NMR titration with LTA caused a concentration-dependent loss of amide peak intensity, and the strongest effects were observed primarily in the R2a subunit, including residues in the RT loop, β3, and the conserved GW motif. This suggests that the R2a subunit contains a major LTA-binding site, a conclusion supported by our NMR titration data and the previously reported A742L mutational data (29). The docked LTA model further supports this interpretation, as residues in the RT loop and nearby β-strand regions form hydrogen-bonding and hydrophobic contacts with the teichoic acid portion of LTA.

The PGN interaction is different from the LTA interaction. PGN binds R2ab with micromolar affinity and is better fitted by a one-site model. NMR CSPs map to residues from both R2a and R2b, forming a cavity between the two subunits. These observations indicate that LTA and PGN do not compete for the same binding surface on R2ab, an arrangement that could allow simultaneous engagement of both cell wall components during autolysin localization. This is consistent with the view that SH3b-like domains are not uniform ligand-binding modules. Some SH3b domains recognize peptide cross-bridges in PGN, as seen in *S. simulans* lysostaphin (48, 58). In others, for example, the bacteriophage PBC5 endolysin, tandem SH3b domains are tightly associated and bind peptidoglycan in a bidentate manner, showing that tandem SH3b domains can act as a single cooperative cell wall recognition unit (59). Other tandem SH3b-like repeats recognize glycan strands or teichoic acid-associated carbohydrate epitopes (59, 60). Therefore, the R2ab repeat expands this theme by showing that a tandem SH3b-like repeat from staphylococcal autolysin can recognize both LTA and PGN, likely through a combination of loop-mediated contacts, charged residues, and the conserved GW-associated binding pockets.

In addition to binding to the cell wall, the R2ab repeat also interacts with abiotic polystyrene surfaces. This surface interaction is central to the proposed role of AtlE in early biofilm formation on materials. Polystyrene is a useful model surface because AtlE-dependent attachment to polystyrene has been described previously (24, 61), and pretreatment of polystyrene surfaces with R2ab reduces *S. epidermidis* biofilm (33). In the present work, we developed paramagnetically spin-labeled PSNPs (PSNP@TEMPO) to identify residue-level information about the orientation of R2ab as it binds to a polystyrene-like surface. The PRE experiment showed that several residues lose intensity in a concentration-dependent manner upon addition of PSNP@TEMPO. Although most residues with strong PRE effects cluster on the C-terminal face of R2b, smaller, scattered effects are also observed on the front faces of R2a and R2b, which may reflect secondary adsorption modes or rearrangement as the nanoparticle surface saturates. ITC analysis also supports a direct interaction between R2ab and PSNP, with submicromolar affinity and a large favorable enthalpic contribution. This is consistent with an interaction that includes electrostatic or polar contacts between R2ab and the negatively charged carboxylate functionalized PSNP surface. The mutational data provide an important functional test of this surface binding model. Pretreatment of polystyrene wells with WT R2ab reduced *S. epidermidis* biofilm formation, whereas the S776A and K779A variants allowed significantly more biofilm formation. These results indicate that nearby polar or charged residues in this region contribute to R2ab adsorption to polystyrene. In contrast, several tyrosine-to-alanine variants inhibited biofilm growth similar to the WT R2ab. However, this finding does not preclude a role for aromatic residues in surface interactions, as alanine substitutions may not fully disrupt the interface's geometry or charge distribution. The more pronounced effects of S776A and K779A suggest that polar and electrostatic interactions probably play a significant role in maintaining stable surface adsorption in these conditions. Together with the PRE mapping, the mutant results support a model in which the R2b C-terminal face contributes to polystyrene binding, while the R2a and interdomain regions remain available for interaction with bacterial cell wall components.

Taken together, these data support a mechanism in which R2ab acts as a molecular bridge during the earliest stage of *S. epidermidis* attachment to abiotic surfaces. In this model, one face of R2ab, centered on the R2a RT loop and GW motif, recognizes LTA embedded in the cell membrane, while a distinct cavity at the R2a/R2b interface accommodates PGN from the cell wall matrix. On the opposing face, a well-defined C-terminal surface of R2b mediates direct polystyrene adsorption, with polar and charged residues, including S776 and K779, making functionally important contributions. Such a functional partitioning would allow AtlE cell-wall recognition on one face and abiotic surface adsorption on the other, thereby providing a structural explanation for how AtlE can simultaneously anchor to the *S. epidermidis* cell wall and adsorb to polystyrene during initial bacterial attachment (Fig. 4*B*). Moreover, the ability of surface-applied R2ab to reduce biofilm formation suggests that blocking or saturating these attachment sites on polystyrene surfaces can interfere with bacterial adhesion. Therefore, the structural and functional data presented here not only define how R2ab engages cell wall components and polystyrene surfaces but also support the idea that disrupting R2ab-mediated attachment may provide a strategy to reduce *S. epidermidis* biofilm formation on medical devices.

## Experimental procedures

### R2ab expression and purification

The R2ab containing pET15b plasmid was transformed into *Escherichia* *coli* BL21 Star (DE3) competent cells (Invitrogen) for expression. In this construct, the R2ab protein is composed of 152 residues with an additional N-terminal 6X His tag for purification. Conditions for optimal over-expression and purification were standardized as described previously (33). For isotopic labeling, R2ab was expressed in M9 minimal media containing ^15^N-ammonium sulfate and ^13^C-glucose (Cambridge Isotope Labs).

### NMR spectroscopy assignments

All samples for NMR experiments were prepared in 50 mM NaCl, 20 mM sodium phosphate (pH 6.5), 6% D_2_O, 0.02% NaN_3_, and protease inhibitor. The final protein concentration was 0.5 mM in a total volume of 550 μl. The experiments were collected on a Bruker Avance III 600 MHz NMR spectrometer equipped with a cryoprobe at Mississippi State University. The following NMR spectra were collected for the resonance assignment of the backbone chemical shifts: ^1^H–^15^N HSQC, ^1^H–^13^C HSQC, HNCA (62), HNCO (63), CBCANH (64), CBCA(CO)NH (65). Non-uniform sampling (NUS) was used to acquire three-dimensional (3D) NMR spectra (66). NMR spectra were processed with NMRPipe, and the NUS spectra were reconstructed by Sparse Multidimensional Iterative Line Shape Enhanced NUS (67, 68). Processed NMR spectra were analyzed using NMRFAM-SPARKY (69) and CARA 1.9.1 (70) to assign the backbone and sidechain chemical shifts. Chemical shift data are deposited to the Biological Magnetic Resonance Data Bank (http://www.bmrb.wisc.edu) under the accession number 30821.

### Solution structure calculations

Distance restraints were obtained from 3D ^15^N-edited NOESY-HSQC spectra (*τ*_mix_ 180 ms and 80 ms) collected at 600 MHz, while ^13^C-edited NOESY-HSQC (*τ*_mix_ 150 ms) and ^13^C aromatic-edited NOESY-HSQC spectra (*τ*_mix_ 150 ms) were collected on a Bruker Avance III 850 MHz NMR spectrometer equipped with a cryoprobe. The NMR spectra were processed with NMRPipe (67), and all NOEs were assigned manually using CARA1.9.1 (70). The torsion angle restraints were obtained from assigned backbone chemical shifts using the program TALOS^+^ (71). The resonance assignment of backbone and sidechain atoms, torsion angles, and NOEs were used in the structural determination with CYANA 2.1 (72). A total of 200 randomized conformers were generated and 20 conformers with the lowest target function, no distance violation >0.5 Å, and no angle violations were selected. These 20 structures were further subjected to molecular dynamics simulation refinement in explicit water with NMR-derived distance and angle restraints using the CNS v1.3 program and the standard water shell refinement protocol (73). The structures were submitted to the Protein Data Bank under accession number 7KWI. PyMOL (74) was used to generate structural figures.

### NMR relaxation measurements

Relaxation measurements were collected for 0.5 mM R2ab ^15^N,^12^C, ^2^H labeled sample following pulse schemes as described by Lakomek *et al.* (75). The T_1_ spin-lattice relaxation rates were recorded with relaxation delays of 0, 20, 60, 100, 200, 400, 600, 800, 1200 ms. The T_2_ relaxation experiments were collected using T_1*ρ*_ NMR experiments with relaxation delays of 0, 2, 12, 24, 48, 72, 96, 120, and 144 ms. The spin lock frequency was set to 2000 Hz for the T_1*ρ*_ experiment. Relaxation rates for both *R*_1_ and *R*_2_ were calculated by fitting the decay intensities from ^1^H–^15^N HSQC spectra. The heteronuclear ^15^N- {^1^H} NOE values were obtained by recording spectra with and without proton presaturation (2 s). All relaxation parameters along with ^15^N-{^1^H} NOE spectra were collected at 25 °C at 600 MHz and 900 MHz field strengths. The R_1_ and R_2_ values were obtained using NMRFAM-SPARKY (69).

### Interaction with cell wall components (LTA and PGN)

Purified *S. aureus* LTA and PGN were purchased from InvivoGen (cat no. tlrl-pslta) and Sigma, respectively. ITC experiments with purified R2ab and LTA or PGN were performed at 25 °C on a VP-ITC calorimeter from MicroCal. The experiments were performed by titrating aliquots of 100 μM R2ab into the sample cell containing 1 mg/ml LTA or PGN in 20 mM sodium phosphate buffer (pH 6.5). The injectant volume was set to 10 μl per injection over 20 s, with a 600 s interval between injections. During the titration, the reaction mixture was continuously stirred at 481 rpm. Control experiments were performed by injecting 100 μM R2ab into the buffer under conditions similar to those used for the LTA and PGN titrations to account for the heat of dilution. The data were analyzed using MicroCal-associated Origin version 7.

To determine the interaction of LTA with the R2ab, ^1^H–^15^N HSQC experiment was done with 100 μM R2ab containing 60 μg or 120 μg LTA in a sample volume of 500 μl in NMR buffer (20 mM sodium phosphate (pH 6.5), 6% D_2_O). A reference experiment with 100 μM R2ab in the absence of LTA was conducted under similar conditions. The spectra were processed using NMRPipe (67) and analyzed by NMRFAM-SPARKY (69). The residue-specific loss in the peak intensity upon binding to the LTA was determined by comparing the peak height of each residue in the absence or presence of LTA. The uncertainty in the peak intensity was determined from Equation 1:

(1) $$\sigma =\frac{I}{I_{0}}\;\sqrt{{\left(\frac{N}{I}\right)}^{2}+{\left(\frac{N_{0}}{I_{0}}\right)}^{2}}$$

where N/I and N_0_/I_0_ represent the inverse of signal to noise ratio in the sample with LTA and without LTA, respectively. Residue showing lower than average plus uncertainty loss in the intensity was considered as significant.

To characterize the interaction of PGN with the R2ab, ^1^H–^15^N HSQC experiment was collected with the ITC sample after the titration between PGN (1 mg/ml) and R2ab (100 μM) was completed. The spectra were processed using NMRPipe (67) and analyzed by NMRFAM-SPARKY (69). The chemical shifts were compared with those of the reference sample of R2ab without PGN in the NMR buffer, as described above. The buffer in both the reference and ITC experiments was identical. The CSP with reference to the sample without PGN was determined from Equation 2:

(2) $${\Delta \delta }_{ppm}=\sqrt{{\left(\Delta H\right)}^{2}+{0.2\left(\Delta N\right)}^{2}}$$

### Docking of LTA and PGN fragments onto R2ab

The docking of LTA and PGN onto R2ab was performed using the Cambridge Crystallographic Data Centre (CCDC) GOLD software (76). The *S. epidermidis* LTA and PGN fragments were prepared, and conformers were subsequently generated using the CSD Conformer Generator. The solution structure of R2ab (PDB ID 7KWI) was used for GOLD docking, and the docking setup was performed using the GOLD docking wizard. A receptor site of 15 × 15 × 15 Å on the R2a domain was defined for the docking of LTA based on the NMR experimental data. Similarly, a receptor site of 15 × 15 × 15 Å on the R2a and R2b domains was defined for the docking of PGN based on the CSP NMR data. During docking of LTA and PGN onto R2ab, the side chains of residues from the R2a subunit, as identified in the NMR LTA titration experiment, and residues showing CSP in the PGN titration were kept flexible. Each ligand conformer was flexibly docked with up to 10 runs using the GOLD genetic algorithm. The docked poses were produced and ranked using the GOLD Chemscore scoring function. The top pose was analyzed and presented in the Results section.

### Isothermal titration calorimetry

For experiments with polystyrene nanoparticles, Polybead carboxylate (polystyrene carboxylate) (0.05 μm) was purchased from Polysciences Inc. Both R2ab and polystyrene nanoparticles were dialyzed in the 20 mM sodium phosphate (pH 6.5) buffer before setting up titration experiments. The carboxylate microspheres were dialyzed at a concentration of 300 nM. The experiments were performed by titrating 100 μM aliquots of R2ab into the sample cell containing 100 nM carboxylate PSNP. The injectant volume was set to 10 μl per injection over 20 s, with a 360 s interval between injections. During the titration, the reaction mixture was continuously stirred at 481 rpm. Control experiments were performed by injecting 100 μM R2ab into the buffer under conditions similar to those used for the LTA and PGN titrations to account for the heat of dilution. The baseline of the raw ITC data was corrected by NITPIC (77), and the data were fit using a nonlinear regression algorithm in the CHASM ITC data analysis program (78).

### Spin-labeling of PSNP and NMR titration

For the PRE experiment with PSNP and R2ab, the carboxylate PSNP was first extensively dialyzed in 50 mM MES buffer pH 5.3. A 500 mM stock of EDC, and sulfo-NHS ester and 100 mM stock of 4-amino-TEMPO spin-label were prepared in MES buffer. First, 20 μl of each EDC and sulfo-NHS ester stock was mixed with 250 μl of 50 mM MES buffer, followed by the addition of 250 μl carboxylate PSNP (300 nM). The reaction tube was kept in the dark for 20 min at RT, followed by the addition of 4-amino-TEMPO in a final concentration of 20 mM. The reaction tube was kept in the dark for 2 h at RT, and excess reactants were removed by extensive dialysis in 20 mM Hepes, pH 6.8. The ^1^H–^15^N HSQC experiment was collected with 100 μM ^15^N R2ab in the absence and presence of PSNP@TEMPO at 10, 20, and 40 nM. The control experiments were done under the same conditions as described above, except that 5 mM ascorbic acid was added to each NMR sample prior to the ^1^H–^15^N HSQC experiment. The spectra were processed using NMRPipe (67) and analyzed by NMRFAM-SPARKY (69). Similar experiments with R2ab (100 μM) and 20 nM PSNP@TEMPO in the presence of 100 and 200 mM NaCl were carried out, including a control experiment containing 5 μM ascorbic acid.

### Biofilm assays

The effects of surface adsorption by WT R2ab and R2ab mutants on *S. epidermidis* biofilm growth were evaluated as described previously (33, 79). In brief, flat-bottomed 96-well polystyrene microtiter plates were coated by adding 50 μM of either bovine serum albumin, WT R2ab, or R2ab mutants (Y722A, S776A, K779A, Y800A, Y843A, and Y844A). Plates were incubated overnight at 4 °C, after which excess protein solution was removed by pipetting. Biofilm formation was initiated by adding 100 μl of *S. epidermidis* strain 1301 (a biofilm-producing strain) to each well in brain heart infusion medium. After static incubation at 37 °C for 24 h, planktonic cells were removed by pipetting, and adherent biofilms were fixed with 100 μl of 100% methanol for 10 min at room temperature. Wells were rinsed with water, and biofilms were stained with 100 μl of 0.1% w/v crystal violet (Thermo Fisher Scientific, B12525). Excess stain was removed by washing three times with PBS buffer, and bound crystal violet was solubilized with 100 μl of glacial acetic acid. Biofilm biomass was quantified by measuring absorbance at 570 nm using a SpectraMax M4 plate reader.

## Data availability

The atomic coordinates and experimental restraints for the solution NMR structure have been deposited in the Protein Data Bank under accession code PDB ID 7KWI. All chemical shifts for backbone, side chain, and NOE assignments have been deposited in the Biological Magnetic Resonance Data Bank (BMRB) under accession number BMRB ID 30821. The underlying ITC and NMR titration and docking data generated during this study are included in the manuscript and its accompanying Supporting Information files. If further information is needed, it may be requested from the corresponding author, Rahul Yadav (rahul.yadav@uafs.edu).

## Supporting information

This article contains supporting information (40, 41, 81, 82).

## Conflict of interest

The authors declare that they have no conflicts of interest with the contents of this article.
