# Supplementary material for: The mechanism of R2ab-mediated attachment to polystyrene surfaces in Staphylococcus epidermidis
Source: J Biol Chem. 2026 Sep 8;302(10):113533. doi: 10.1016/j.jbc.2026.113533 (PMC13677396; doi:10.1016/j.jbc.2026.113533)
Supplement: Supplementary file 1 [file mmc1.docx]

*Supporting Information for:*

**The Mechanism of R2ab-mediated Attachment to Polystyrene Surfaces in *Staphylococcus epidermidis***

Rahul Yadav,^1,2*^ Y. Randika Perera,^2^ Radha P. Somarathne,^2^ Fnu Himanshi,^1^ Soneika Carby,^1^ Archana Mishra,^1^ and Nicholas C. Fitzkee^2*^

^1^Physical Science Department, University of Arkansas-Fort Smith, Fort Smith, AR, USA

^2^Department of Chemistry, Mississippi State University, Mississippi State, MS 39762, USA

^*^To whom correspondence should be addressed.

Email: [rahul.yadav@uafs.edu](mailto:rahul.yadav@uafs.edu); [nfitzkee@chemistry.msstate.edu](mailto:nfitzkee@chemistry.msstate.edu)

Contents

[Supplementary Materials and Methods	2](#_Toc232260304)

[Supplementary Tables	3](#_Toc232260305)

[Table S1: Resonance assignment statistics for R2ab..	3](#_Toc232260306)

[Table S2: Structural parameters of the R2ab solution structure.	4](#_Toc232260307)

[Table S3: The average ^15^N relaxation rates and model-free parameters of R2ab.	5](#_Toc232260307)

[Supplementary Figures	6](#_Toc232260309)

[Figure S1: ^1^H-^15^N HSQC spectrum of R2ab.	6](#_Toc232260310)

[Figure S2: Overlay of solution NMR and crystal structures of R2ab.	7](#_Toc232260311)

[Figure S3: Fast (ps-ns) and slow (μs-ms) backbone dynamics in R2ab.	8](#_Toc232260312)

[Figure S4: Denaturation profiles of R2ab.	9](#_Toc232260313)

[Figure S5: Characterization of LTA binding to R2ab.	10](#_Toc232260314)

[Figure S6: Characterization of PGN binding to R2ab.	11](#_Toc232260315)

[Figure S7: Physicochemical characterization of PSNP.	12](#_Toc232260316)

[Figure S8. HSQC spectrum of R2ab with spin-labeled PSNP@TEMPO.	13](#_Toc232260317)

[Figure S9. Polystyrene microtiter plate biofilm assay of *S. epidermidis*……………………………….13](#_Toc232260317)

[References	15](#_Toc232260323)

# Supplementary Materials and Methods

**Chemical and thermal unfolding**

The Gibbs energy of unfolding in buffer 𝛥G° (H_2_O) at varying temperatures was extrapolated using denaturation experiments performed with guanidinium chloride (GdmCl). Folding was monitored using Trp fluorescence on a Horiba FluoroMax 4 spectrofluorometer equipped with a Quantum Peltier-based cuvette holder and a Hamilton Microlab dual syringe auto-titrator for all measurements (λ_ex_= 280nm, λ_em_=300-400 nm). A Sterna 1 cm x 1 cm path length rectangular quartz cuvette with a small magnetic stir bar was used for mixing. Spectra are the average of a series of 5 scans made at 1-nm intervals, 0.1 second signal integration time, and 5.0 nm entrance and exit slit bandpass. All spectra have been baseline corrected using the appropriate blank (buffer plus GdmCl). The titrant consisted of 6.0-7.0 M GdmCl, and final GdmCl concentrations were determined using an Abbe refractometer (1). The auto-titrator was loaded with 5 mL of GdmHCl and programmed for 24 titration steps, starting at 0.1 M and ending at 4.0 M GdmHCl. The sample was equilibrated with mixing for 300 seconds before the spectra were recorded between titration steps. Titrations were completed at five temperatures in triplicate, 15 °C, 20 °C, 25 °C, 30 °C, and 35 °C. The maximum emission wavelength for folded R2ab was 343 nm, and unfolding curves at each temperature were generated and fit to a two-state model. A Van’t Hoff plot was generated and Δ*H*_VH_ calculated.

# Supplementary Tables

## Table S1: Resonance assignment statistics for R2ab

| ***Resonance Assignment*** | ***% Completion*** |
| --- | --- |
| Cα | 95 |
| Cβ | 95 |
| C' | 95 |
| Non-aromatic and non-carboxyl side-chain carbons | 78 |
| Aromatic side chain carbon | 53 |
| Hα | 96 |
| Hβ | 96 |

Unassigned main chain residues: N696, N697, N703, N730, Q731, T806, D824, and K825).

## Table S2: Structural parameters of the R2ab solution structure

| ***Distance Restraint List*** | |
| --- | --- |
| Sequential | 372 |
| Intra-residue | 594 |
| Medium-range | 453 |
| Long-range | 112 |
| Hydrogen bonds | 47 |
| Dihedral angle restrains (phi and psi) | 260 |
| ***RMSD Values for Ordered Residues^#^*** | |
| All backbone atoms | 1.0 Å |
| All heavy atoms | 1.5 Å |
| ***Ramachandran Plot Statistics*** | |
| Most favored region (%) | 82.3% |
| Allowed region (%) | 16.3% |
| Additionally allowed region (%) | 0.7% |
| Disallowed region (%) | 0.8% |
| ^#^698-713, 718-222, 724-730, 733-797, 799-801, 803-807, 810-846 | |

## Table S3: The average ^15^N relaxation rates and model-free parameters of R2ab

|  | **600 MHz** | **900 MHz** |
| --- | --- | --- |
| {^1^H}–^15^N heteronuclear NOE | 0.79 ± 0.07 | - |
| Longitudinal relaxation rates (*R*_1_) | 1.05 ± 0.01 s^−1^ | 0.78 ± 0.02 s^−1^ |
| Transverse relaxation rates (*R*_2_) | 14.2 ± 0.1 s^−1^ | 18.3 ± 0.2 s^−1^ |
| *R*_2_/*R*_1_ | 13.9 ± 0.2 | 24.3 ± 0.6 s^−1^ |
| **Model-Free parameters** | | |
| Order parameter (*S*^2^) | 0.80 ± 0.01 | |
| Effective rotational correlation time (𝜏_c_) | 11.3 ns | |
| Isotropic rotational correlation time (𝜏_m_) | 11.1 ns | |
| Diffusion model | axially symmetric anisotropic diffusion | |
| Isotropic Diffusion Coefficient (D_iso_) | 1.5 x 10^7^ s^-1^ | |
| Axially Symmetric Anisotropy (D_a_) | 1.3 x 10^7^ s^-1^ | |
| Diffusion anisotropy ratio, D_ratio_ (D_॥_/D_⊥_) | 0.029 | |

# Supplementary Figures

## Figure S1: ^1^H-^15^N HSQC spectrum of R2ab. The HSQC spectrum of ^15^N-labeled R2ab (0.5 mM) was recorded at 298 K at 600 MHz in 6% D_2_O, 20 mM sodium phosphate buffer, 50 mM NaCl, pH 6.5. The cross-peaks are labeled with single-letter amino acid residue names and sequence numbers. The inset shows the labeling of the central crowded region.

## Figure S2: Overlay of solution NMR and crystal structures of R2ab. Comparison of the R2ab crystal structure (PDB ID: 4EPC) with the solution NMR structure of R2ab (PDB ID: 7KWI) highlights structural differences, primarily in the RT loop and distal loop of the R2a subunit, as well as the distal loop and SU loop of the R2b subunit. The residues comprising the GW motifs in the R2a and R2b subunits are shown as sticks and are labeled.

## Figure S3: Fast (ps-ns) and slow (μs-ms) backbone dynamics in R2ab. *A*, Plots of optimized model-free parameters: the effective correlation time (𝜏_e_) and the chemical exchange parameter (*R*_ex_), as a function of residue number. *B*, The R2ab structure is displayed with the axis of the ellipsoid diffusion tensor determined from the model-free analysis in the Relax Program (2). Both domains of R2ab rotate as a single unit, with an effective rotational correlation time (𝜏_c_) of 11.3 ns. The fully anisotropic diffusion model was selected by Akaike’s Information Criteria (3)

## Figure S4: Denaturation profiles of R2ab. *A*, thermal denaturation using differential scanning calorimetry (DSC). Open circle and continuous line represent the experiment and the two-state fit, respectively. *B*, Guanidinium hydrochloride denaturation curves for R2ab. Temperatures are shown in the inset. Curves represent two-state unfolding curves using the linear extrapolation model after baseline subtraction. *C*, Van’t Hoff plot constructed from the data in panel B for R2ab chemical denaturation. Error bars represent the propagated standard uncertainty derived from three independent replicates.

## Figure S5: Characterization of LTA binding to R2ab. *A*, overlay ^1^H-^15^N HSQC spectra of R2ab (100 µM) in the absence (red) or presence of LTA (blue,60 µg in 500 µl sample; green, 500 µg in 500 µl sample). The loss of amide peak intensity in the presence of LTA was observed without any chemical shift perturbation. *B*, The cartoon representation of R2ab shows LTA binding site residues (highlighted as stick model) based on the peak intensity reduced by the mean and two standard deviations. *C,* the LigPlot^+^ (4) model of the R2ab and LTA interaction highlights the residues of R2ab that are involved in electrostatic and hydrophobic interactions with the LTA.

## Figure S6: Characterization of PGN binding to R2ab. *A*, overlay of ^1^H-^15^N HSQC spectra of R2ab (100 µM) in the absence (red) or presence of PGN (blue). Residues showing CSP are labeled in red. *B*, Chemical Shift Perturbation (CSP) of R2ab upon PGN binding was calculated from spectra in 6A and plotted against residue number. A CSP cutoff of 0.02 ppm, indicated by a red dashed line, was considered significant. The residues showing significant CSP are labeled.

## Figure S7: Physicochemical characterization of PSNP. Hydrodynamic diameter, polydispersity, and zeta potential of carboxyPSNP and PSNP@TEMPO. Data are presented as mean ± standard deviation of three independent replicates.

## Figure S8. HSQC spectrum of R2ab with spin-labeled PSNP@TEMPO. *A*, Overlay of ^1^H-^15^N HSQC of 100 µM R2ab (black) in the absence and presence of spin-labeled PSNP@TEMPO (10, 20, and 40 nM). *B*, the intensity ratio (I/I_0_) of the 100 µM R2ab in the absence and the presence of spin-labeled PSNP@TEMPO (10, 20, and 40 nM).

**Figure S9. Polystyrene microtiter plate biofilm assay of *S. epidermidis***. The wells of the polystyrene microtiter plates were preincubated with proteins (BSA, R2ab, or R2ab mutants) for 1 hr before *S. epidermidis* was added and then grown at 37 °C for 48 hr in BHI media. The plates were stained with crystal violet, and the biofilms formed were dissolved in 20% acetic acid, and the optical density at OD_570_ was measured.

# References

1.	Pace, C. N. (1986) Determination and analysis of urea and guanidine hydrochloride denaturation curves. *Methods Enzymol.* **131**, 266–280

2.	Bieri, M., d'Auvergne, E. J., and Gooley, P. R. (2011) relaxGUI: a new software for fast and simple NMR relaxation data analysis and calculation of ps-ns and mus motion of proteins. *J. Biomol. NMR* **50**, 147–155

3.	d'Auvergne, E. J., and Gooley, P. R. (2003) The use of model selection in the model-free analysis of protein dynamics. *J. Biomol. NMR* **25**, 25–39

4.	Laskowski, R. A., and Swindells, M. B. (2011) LigPlot+: multiple ligand-protein interaction diagrams for drug discovery. *J Chem Inf Model* **51**, 2778–2786
